# Somatic mosaicism in *STAG2*-associated cohesinopathies: Expansion of the genotypic and phenotypic spectrum

**DOI:** 10.3389/fcell.2022.1025332

**Published:** 2022-11-16

**Authors:** Julia Schmidt, Steffi Dreha-Kulaczewski, Maria-Patapia Zafeiriou, Marie-Kristin Schreiber, Bernd Wilken, Rudolf Funke, Christiane M Neuhofer, Janine Altmüller, Holger Thiele, Peter Nürnberg, Saskia Biskup, Yun Li, Wolfram Hubertus Zimmermann, Silke Kaulfuß, Gökhan Yigit, Bernd Wollnik

**Affiliations:** ^1^ Institute of Human Genetics, University Medical Center Göttingen, Göttingen, Germany; ^2^ Department of Pediatics and Adolescent Medicine, University Medical Center Göttingen, Göttingen, Germany; ^3^ Institute of Pharmacology and Toxicology, University Medical Center Göttingen, Göttingen, Germany; ^4^ DZHK (German Center for Cardiovascular Research), Partner Site Göttingen, Göttingen, Germany; ^5^ Cluster of Excellence “Multiscale Bioimaging: from Molecular Machines to Networks of Excitable Cells” (MBExC), University of Göttingen, Göttingen, Germany; ^6^ Department of Pediatric Neurology, Klinikum Kassel, Kassel, Germany; ^7^ Institute of Neurogenomics, Helmholtz Zentrum Munich, Munich, Germany; ^8^ Department of Neurology, Friedrich-Baur-Institute, LMU Hospital, Ludwig Maximilians University, Munich, Germany; ^9^ Cologne Center for Genomics (CCG), University of Cologne, Faculty of Medicine, University Hospital Cologne, Cologne, Germany; ^10^ Berlin Institute of Health at Charité, Core Facility Genomics, Berlin, Germany; ^11^ Max Delbrück Center for Molecular Medicine in the Helmholtz Association, Berlin, Germany; ^12^ CeGaT GmbH, Center for Genomics and Transcriptomics, Tübingen, Germany

**Keywords:** STAG2, cohesinopathies, mosaic disorders, supernumerary nipples, bioengineered neuronal organoids

## Abstract

STAG2 is a component of the large, evolutionarily highly conserved cohesin complex, which has been linked to various cellular processes like genome organization, DNA replication, gene expression, heterochromatin formation, sister chromatid cohesion, and DNA repair. A wide spectrum of germline variants in genes encoding subunits or regulators of the cohesin complex have previously been identified to cause distinct but phenotypically overlapping multisystem developmental disorders belonging to the group of cohesinopathies. Pathogenic variants in *STAG2* have rarely been implicated in an X-linked cohesinopathy associated with undergrowth, developmental delay, and dysmorphic features. Here, we describe for the first time a mosaic *STAG2* variant in an individual with developmental delay, microcephaly, and hemihypotrophy of the right side. We characterized the grade of mosaicism by deep sequencing analysis on DNA extracted from EDTA blood, urine and buccal swabs. Furthermore, we report an additional female with a novel *de novo* splice variant in *STAG2*. Interestingly, both individuals show supernumerary nipples, a feature that has not been reported associated to *STAG2* before. Remarkably, additional analysis of *STAG2* transcripts in both individuals showed only wildtype transcripts, even after blockage of nonsense-mediated decay using puromycin in blood lymphocytes. As the phenotype of *STAG2*-associated cohesinopathies is dominated by global developmental delay, severe microcephaly, and brain abnormalities, we investigated the expression of *STAG2* and other related components of the cohesin complex during Bioengineered Neuronal Organoids (BENOs) generation by RNA sequencing. Interestingly, we observed a prominent expression of *STAG2*, especially between culture days 0 and 15, indicating an essential function of *STAG2* in early brain development. In summary, we expand the genotypic and phenotypic spectrum of *STAG2*-associated cohesinopathies and show that BENOs represent a promising model to gain further insights into the critical role of STAG2 in the complex process of nervous system development.

## 1 Introduction

The *STAG2* gene (MIM 300826, NM_001042750) is located on Xq25 and comprises 35 different exons. Its gene product is a component of the large, evolutionarily highly conserved cohesin complex, which has been linked to various cellular processes like genome organization, DNA replication, gene expression, heterochromatin formation, sister chromatid cohesion, and DNA repair (Mehta et al., 2013; Mullegama et al., 2017; Chu et al., 2022). SMC1A, SMC3, RAD21, and either STAG1 or STAG2 form four core subunits of the cohesin complex (Yuan et al., 2019). The function of the cohesin complex is regulated by several additional proteins including NIPBL, ESCO2, HDAC8, DDX11, SGOL1, WAPL, PDS5A, PLK1, AURKB, and ATRX (Sumara et al., 2002; Terret et al., 2009; Kernohan et al., 2010; van der Lelij et al., 2010; Kim et al., 2013; Chetaille et al., 2014; Kaiser et al., 2014; Alomer et al., 2017; Parenti et al., 2020; Liu et al., 2021). A wide spectrum of germline variants in genes encoding either these regulators or core subunits of the cohesin complex have previously been identified to cause distinct multisystem developmental disorders belonging to the group of cohesinopathies, including Cornelia de Lange syndrome (CdLS), Roberts/SC phocomelia syndrome (RBS), α-thalassemia/mental retardation syndrome (ATRX), Warsaw breakage syndrome (WBS), and chronic atrial and intestinal dysrhythmia (CAID) (McNairn and Gerton, 2008; Gerkes et al., 2010; Barbero, 2013; Ball et al., 2014; Mullegama et al., 2017). These cohesin-associated disorders have overlapping phenotypic characteristics manifesting in various combinations of the following features: developmental delay, pre- and postnatal growth retardation, microcephaly, limb reduction defects, and dysmorphic features. In line with the cellular processes in which the cohesin complex is involved in, like, e.g., repair of DNA double-strand breaks, postzygotic pathogenic variants affecting its function are frequently found in cancer (Sarogni et al., 2020). The best characterized cohesinopathy is CdLS, an autosomal dominant (*NIPBL, SMC3,* and *RAD21*) or X-linked (*SMC1A* and *HDAC8*) malformation syndrome (Krantz et al., 2004; Tonkin et al., 2004; Musio et al., 2006; Deardorff et al., 2007; Deardorff et al., 2012a; Deardorff et al., 2012b; Harakalova et al., 2012). CdLS patients are small in size and have a characteristic facial appearance including thick eyebrows, synophrys, a short nose with an upturned nasal tip, a long and smooth philtrum and a thin upper lip (Kline et al., 2018). The majority of CdLS cases is caused by variants in *NIPBL* (Chandrasekaran et al., 2022) with 15% of them predicted to affect splicing (Mannini et al., 2013). Interestingly, somatic mosaicism is frequently (15%–20%) seen in individuals with classic features of CdLS (Krawczynska et al., 2018). In general, identification of mosaic variants can be challenging as they may not be detectable in genomic DNA from standard peripheral blood samples, but only in affected tissue. Thus, a specific clinical suspicion is often required to select the appropriate tissue and to detect the causative variant. Germline variants in *STAG2* have been described to cause an X-linked cohesinopathy associated with growth retardation, developmental delay, and dysmorphic features, both in females and males (Mullegama et al., 2017; Mullegama et al., 2019). The phenotypic severity depends on the type and localization of the variant as well as the X-chromosome inactivation pattern (Mullegama et al., 2017; Kruszka et al., 2019; Mullegama et al., 2019). So far, mosaic *STAG2* variants have not been described in individuals with multisystem anomalies and neurodevelopmental delay. As *STAG2*-related disorders generally seem to be at the milder end of the cohesinopathy spectrum, a specific clinical diagnosis can be challenging (Yuan et al., 2019). According to the literature (HGMD Database 2022.2), 26 pathogenic *STAG2* variants (eight nonsense variants, five missense variants, three frameshift variants, one splice variant and nine gross insertions) have been reported to date in patients with intellectual disability and congenital anomalies (Figure 3D).

Here, we describe for the first time a mosaic *STAG2* variant (c.2184=/G>T p.Gln728=/His) in an individual with developmental delay, microcephaly, and hemihypotrophy of the right side. Furthermore, we report an additional female with a novel *de novo* splice variant in *STAG2* (c.1412_1416+9del p.?). Interestingly, both individuals reported here show supernumerary nipples (the mosaic individual only on the affected side), a feature which has not been reported to be associated to pathogenic *STAG2* variants before. To further determine the role of STAG2 during early brain development, we performed RNA sequencing to investigate the expression of *STAG2* and other cohesin complex components, using Bioengineered Neuronal Organoids (BENOs), which have proven to be a promising tool to gain further insights into the development of the nervous system in health and disease (Zafeiriou et al., 2020; Islam et al., 2021; Wrobel et al., 2021). We were able to show that *STAG2* is expressed at very early stages during BENO generation, suggesting a critical role of *STAG2* in brain development.

## 2 Material and methods

### 2.1 Genetic analysis

Written informed consent was obtained from all participants or their legal representatives prior to participation in the study. The study was approved by the Ethics Committee of University Medical Center Göttingen (approval number 3/2/16) and performed in accordance with the Declaration of Helsinki protocols. DNA isolation from EDTA blood was carried out following standard protocols for all participants. In family 1, we performed exome sequencing (ES) for individual 1 [II-2, Figure 3A (left panel)]. Sequencing libraries were prepared using the Twist enrichment workflow (Twist Bioscience, San Francisco, CA) and the Twist Core exome kit with spiked-in Twist RefSeq probes as well as a custom-design spike-in to enrich mitochondrial DNA. Library preparation and capture was performed according to the manufacturer’s instructions and paired-end sequencing was performed on a NovaSeq 6000 instrument (Illumina, San Diego, CA). Sanger sequencing was used to validate the NGS data and to test the parents (Supplementary Table S1). To further analyze the grade of mosaicism, we performed deep sequencing analysis on PCR products including the mutated region of *STAG2* obtained from DNA extracted from EDTA blood, urine and buccal swabs. In family 2, we performed ES on DNA extracted from blood of individual 2 [II-2, Figure 3A (right panel)] and her parents (I-1 and I-2), using the Agilent SureSelect Human All Exon V6 enrichment kit (Agilent Technologies, Santa Clara, CA) and the Illumina HiSeq4000 sequencer (Illumina, San Diego, CA). ES data were analyzed using Varbank 2.26 (Cologne Center for Genomics, University of Cologne, Cologne, Germany) based on gnomAD Exomes + Genomes 2.1.1, dbSNP 142 and ClinVar 202,109. Sanger sequencing was used to validate the NGS data (Supplementary Table S1).

### 2.2 Sequencing of *STAG2* RNA and qPCR analysis

RNA from peripheral blood lymphocytes from both patients as well as a wildtype control was collected using PAXGene blood RNA Tubes and isolated according to manufacturer’s instructions by using PAXgene Blood RNA kit (PreAnalytix GmbH, Hombrechtikon, Switzerland). For treatment of blood lymphocytes with puromycin, fresh peripheral blood samples of both individuals were cultured in LymphoGrow II medium (Cytogen, Greven, Germany) for 5 days. Before harvesting cells by centrifugation, a treatment with puromycin (final concentration 200 μg/ml in culture medium) for 4 h was performed. RNA was extracted from cells as mentioned above. Four different primer pair combinations were used to amplify the specific region between exons 12 and 18 (individual 2) and exons 20 and 24 (individual 1) (Supplementary Table S1). All PCR products were sequenced on an ABI3500 and analysed using SeqPilot Software 5.2.0 (JSI medical systems GmbH, Ettenheim, Germany). Quantitative RT-PCR was performed using SYBR Green (QIAGEN, Hilden, Germany) on a QuantStudio 5 (ThermoFisher Scientific Inc., Waltham, United States) in two biological replicates for *STAG2* and *ACTB* as housekeeping gene, and each measurement was performed in triplicates (Supplementary Table S1). Relative *STAG2* expression was calculated using ddCT method.

### 2.3 Gene expression studies in bioengineered neuronal organoids

Bioengineered neuronal organoids (BENOs) have been generated from the induced pluripotent stem cell line TC1133 (Baghbaderani et al., 2015) using the differentiation protocol as previously described (Zafeiriou et al., 2020). RNA was extracted from BENOs on culture days 0, 3, 8, 15, 28, 40, 50, 60, and 90 using the Macherey-Nagel isolation kit (cat. no. 740955), according to the manufacturer’s instructions. Samples d-0-d-60 were prepared as described previously (Zafeiriou et al., 2020) while for d-90 bcl files were demultiplexed and converted to fastq using bcl2fastq v2.20. The sequencing quality was asserted using FastQC (http://www.bioinformatics.babraham.ac.uk/projects/fastqc/). Sequences were aligned to the reference genome GRCh38.p13 using the RNA-Seq STAR alignment tool (Dobin et al., 2013) followed by quantification using RSEM (version 1.3.1) (Li and Dewey, 2011). Estimated gene expression levels were analyzed in the R/Bioconductor environment (version 4.1.0) using the DESeq2 package version 1.32.0 (Love et al., 2014). Candidate genes were filtered using an absolute log2 fold-change > 1 and FDR-corrected *p*-value < 0.05. For data visualization, normalized FKPM values (DESeq2) of genes of interest (*STAG2*, *SMC1A*, *SMC3*, *RAD21*, *NIPBL,* and *BRD4*) were extracted and displayed in GraphPad Software (Prism 9.1). Further details can be found in Supplementary Table S2.

## 3 Results

### 3.1 Clinical reports

#### 3.1.1 Individual 1

**FIGURE 1:**
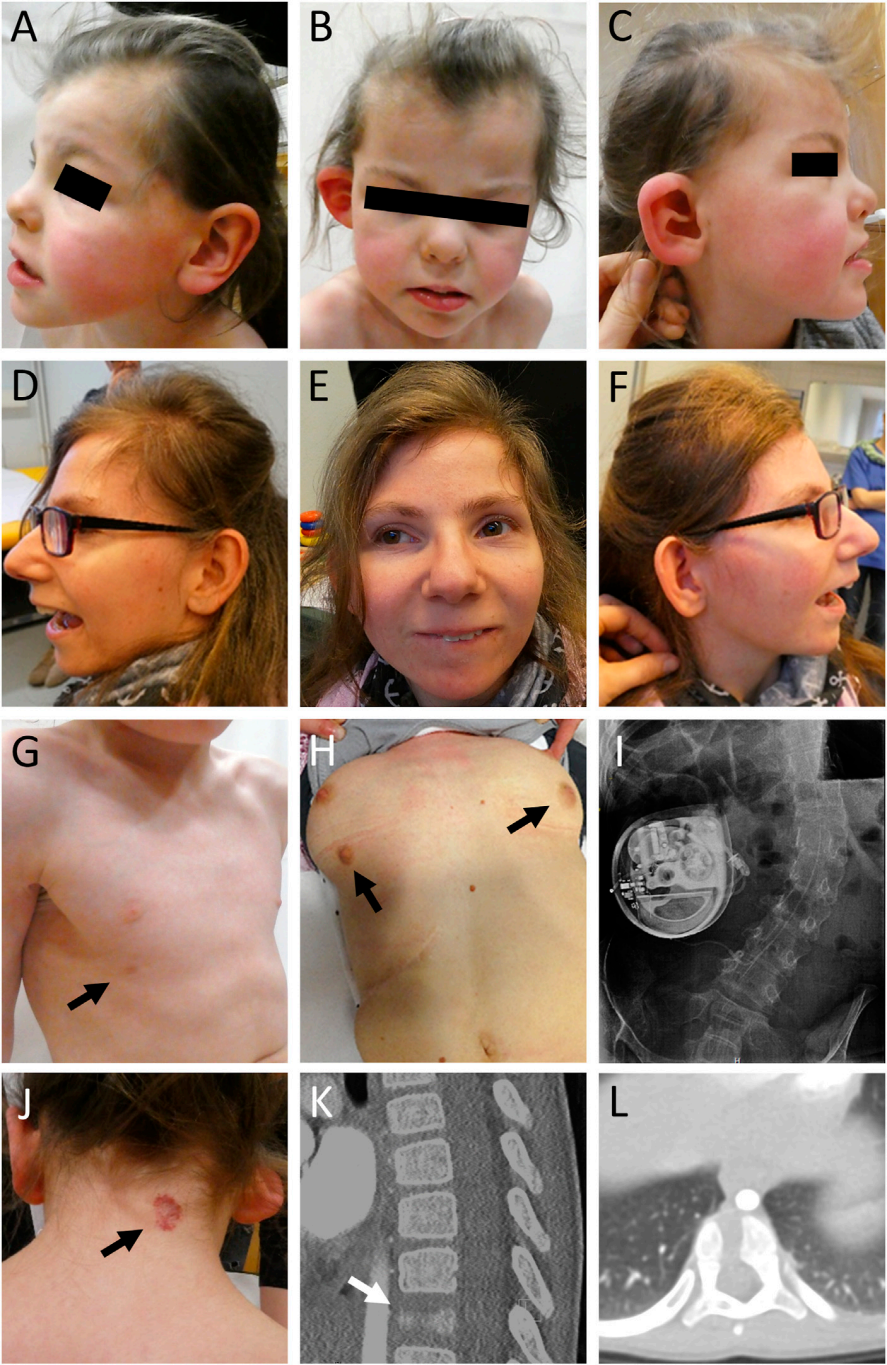
Clinical pictures and radiographs of affected individuals. **(A–C)** Clinical characteristics of individual 1 included microcephaly, facial asymmetry, widow’s peak, slightly low-set and posteriorly rotated ears, a protruding dysplastic right ear, prominent eyebrows, a wide nasal bridge, and a thin upper lip. **(D–F)** Individual 2 showed severe microcephaly, slightly low-set and posteriorly rotated ears, prominent eyebrows, strabismus, a prominent nose, a thin upper lip, and retrognathia. **(G)** Individual 1 showed a supernumerary nipple only on the right side (indicated by black arrow). **(H)** Individual 2 showed bilateral supernumerary nipples and breasts (indicated by black arrows) as well as **(I)** scoliosis. She received treatment for dystonia with a baclofen pump. **(J)** In addition, individual 1 presented with a low posterior hairline, a hemangioma (indicated by black arrow) on the neck, **(K,L)** and a butterfly vertebra Th11 (indicated by white arrow).

**FIGURE 2:**
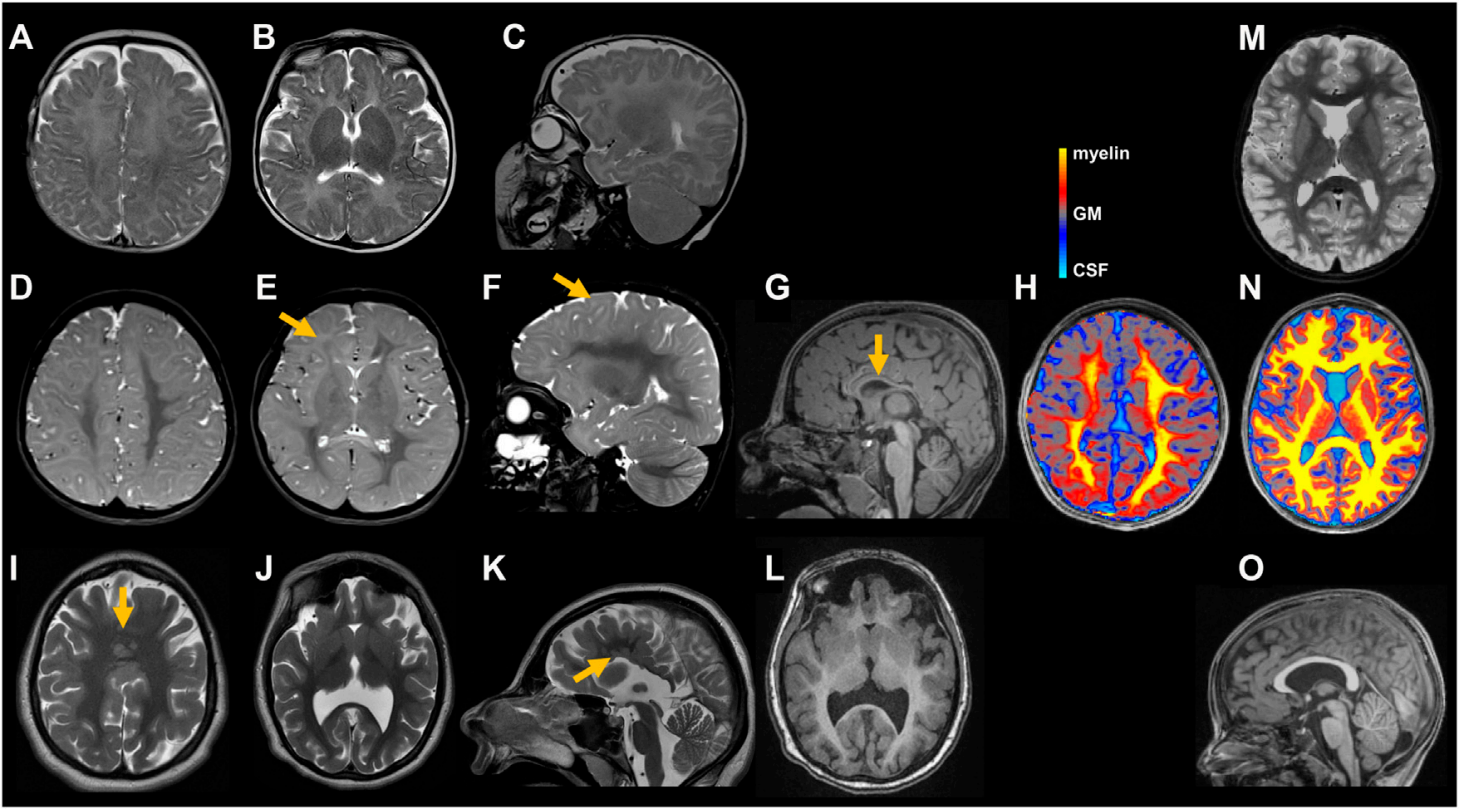
Cranial images of individuals with *STAG2*-associated cohesinopathy. Individual 1 underwent MRIs at age 5 months **(A–C)** and 5 years and 4 months **(D–G)**. Minor delay of myelination can be seen on the initial axial **(A,B)** and sagittal **(C)** T2 weighted (T2w) images. At 5 years and 4 months, myelination is still incomplete as illustrated in axial **(D,E)** and sagittal **(F)** T2w images affecting peripheral white matter regions [indicated by yellow arrow in **(E)**] and U-fibers [indicated by yellow arrow in **(F)**]. Right hemisphere appears hypotrophic **(D,E)**. T1 weighted (T1w) sagittal image **(G)** shows a thin and dysplastic corpus callosum [indicated by yellow arrow in **(G)**]. Axial magnetization transfer saturation (MTsat) map **(H)**: Semi-quantitative MRI marker for myelin depicts a myelin deficit, pronounced in peripheral white matter and U-fibers. Color scale represents MTsat values with highest values (yellow) found in myelin and gradually decreasing to gray matter (GM) (grey) and cerebrospinal fluid (CSF) (turquoise). For detailed description of MR methods see (Dreha-Kulaczewski et al., 2012) **(H)**. MRI of individual 2 at age 17 years and 2 months reveals normal myelination but profound malformations including multiple periventricular nodular gray matter heterotopias and a semilobar holoprosencephaly as illustrated in axial **(I,J)** and sagittal **(K)** T2w images as well as axial T1w image **(L)** including multiple periventricular nodular gray matter heterotopias [indicated by yellow arrow in **(K)**] and a semilobar holoprosencephaly [indicated by yellow arrow in **(I)**]. Cranial images from age-matched healthy individuals **(M–O)**. Axial T2w images **(M)** shows fully myelinated white matter including the subcortical U-fibers. MTsat map **(N)** illustrates uniformly high values (yellow) in the white matter. Sagittal T1w image **(O)** displays a normal shaped corpus callosum.

**FIGURE 3:**
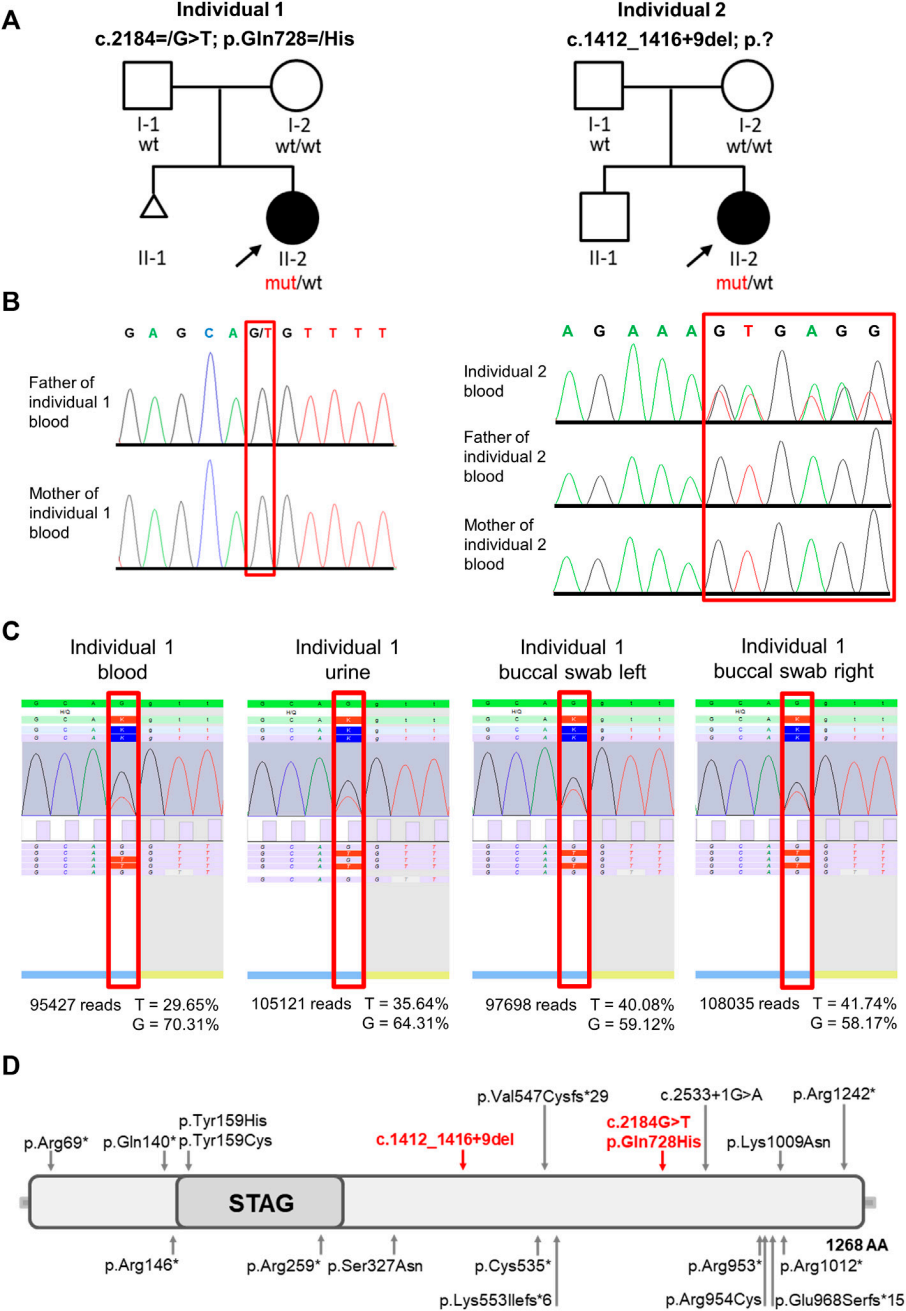
Pedigrees, molecular findings, structure of STAG2 protein and location of the identified variants. **(A)** Pedigrees of the 2 families included in this study. Affected and unaffected individuals are indicated by filled and open squares (males) and circles (females), respectively. **(B)** Electropherograms show that the *STAG2* variant c. 2184G>T p.(Gln728>His) is not detectable in blood samples of both unaffected parents of individual 1 and that the heterozygous *STAG2* variant c.1412_1416+9del (p.?) in individual 2 is not detectable in blood samples of both unaffected parents of individual 2. **(C)** Deep sequencing analysis was used to validate the exome data and confirmed the mosaic *STAG2* variant c.2184=/G>T p.(Gln728=/His): The variant was present in 29.65% of the 95,427 reads from the EDTA blood sample, in 35.64% of the 105,121 reads from urine, in 40.08% of the 97,698 reads from the buccal swab (left side) and in 41.74% of the 108,035 reads from the buccal swab (right side). **(D)** Identified variants and schematic overview of their location within the STAG2 protein. Red arrows point to the locations of the 2 variants identified in this study, previously reported variants are indicated by grey arrows.

The girl was born to unrelated healthy parents at 39 weeks of gestation by C-section after unremarkable pregnancy. Birth weight was 3,400 g (+0.1 SD), length 51 cm (−0.05 SD) and OFC 34 cm (−0.5 SD). Dysplasia of the right ear combined with hearing impairment on the right side as well as facial nerve paralysis on the left side and a hemangioma located on the neck were noted shortly after delivery (Figure 1). At the age of 5 months, she had a first seizure and she continued to have generalized tonic-clonic seizures regularly. Her development was delayed, she walked without support at 25 months of age and started to speak first words at the age of 2 years. She received occupational therapy, physiotherapy, and speech therapy. Due to a moderate hearing impairment on the right side, she received a unilateral hearing aid. At clinical evaluation at 5 years and 2 months, height was 108 cm (−0.9 SD), weight 15.9 kg (−1.5 SD), and head circumference was 45 cm (−5.3 SD). She showed a widow’s peak, a low posterior hairline, slightly low-set and posteriorly rotated ears, a protruding dysplastic right ear, prominent eyebrows, a wide nasal bridge, deep-set eyes with dark eyelashes, a limbal dermoid at the right eye, and a thin upper lip. Additionally, she had a hemangioma on the neck, a supernumerary nipple on the right side, bilateral single transverse palmar crease, hypertrichosis, scoliosis, vertebral abnormalities including “butterfly” vertebrae and hemi-vertebrae, and a general hemihypotrophy of the right side. Her gait was ataxic. Cranial MRI (cMRI) at the age of 5 months revealed a minor delay of myelination (Figures 2A–C). At 5 years and 4 months myelination was still incomplete affecting peripheral white matter regions and U-fibers (Figures 2D–F). Right hemisphere appeared hypotrophic (Figures 2D,E). The corpus callosum was thin and dysplastic (Figure 2G). Magnetization transfer saturation map confirmed the myelin deficit, pronounced in peripheral white matter and U-fibers (Figure 2H). Cranial images from age-matched healthy individuals (Figures 2M,N). The mother had one early miscarriage, otherwise family history was unremarkable (Figure 3A).

#### 3.1.2 Individual 2

Individual 2 was born at 39 weeks of gestation to healthy parents. Birth weight was 3,140 g (−0.5 SD), length 47 cm (−1.9 SD) and OFC 31 cm (−2.8 SD). Developmental delay was noted shortly after birth. She started to speak first words at the age of 4 years. So far, she has not acquired independent walking skills. At clinical evaluation at 24 years, height was 133 cm (−7.0 SD), weight 33 kg (−7.3 SD), and head circumference was 48 cm (−6.0 SD). She showed severe microcephaly, slightly low-set and posteriorly rotated ears, prominent eyebrows, strabismus, a prominent nose, a thin upper lip, retrognathia, bilateral supernumerary nipples and breasts, scoliosis, severe generalized dystonia, and spasticity. cMRI studies revealed a frontal fusion of the hemispheres as well as a hypoplastic corpus callosum with partial agenesis in the corpus region and heterotopic cortical tissue in these areas (Figures 2I–L). Cranial image from age-matched healthy individual (Figure 2O). She received treatment for dystonia with a baclofen pump. The family history was unremarkable (Figure 3A).

### 3.2 Molecular findings

Initial genetic testing including conventional cytogenetic analysis and array CGH was unremarkable for both individuals. Therefore, exome sequencing (ES) was performed on DNA extracted from peripheral blood of individual 1 [II-2, Figure 3A (left panel)]. After in depth filtering and variant interpretation, we identified a mosaic *STAG2* variant NM_001042749.2:c.2184=/G>T p.(Gln728=/His) in 20% of reads (42 of 209 reads). The variant was not present in the gnomAD database (https://gnomad.broadinstitute.org) [last access date 10/08/2022, (Karczewski et al., 2020)] and predicted to severely impact on protein function. The variant, c.2184=/G>T, is located in exon 22 of the *STAG2* gene and leads to the substitution of a glutamine at the amino acid position 728 by histidine (p.Gln728His). The variant was predicted as disease-causing by MutationTaster (http://www.mutationtaster.org), damaging by SIFT (https://sift.bii.a-star.edu.sg) and damaging by M-CAP (http://bejerano.stanford.edu/MCAP/), indicating deleteriousness of this variant. The CADD PHRED score is 36. The variant c.2184G>T p.(Gln728His) affects a moderately conserved amino acid. Glutamine is present in 10/13 species (Alamut Visual Plus software v.1.6.1). As the mutated base is located at the very end of exon 22 of *STAG2* transcript *in silico* splice prediction tools predict an aberrant splicing [SpliceAI: splice-altering (0.98); RF: deleterious (1); scSNV ADA: deleterious (1)]. Sanger sequencing was used to test the parents. We could not detect the variant in the DNA extracted from peripheral blood lymphocytes of both unaffected parents (Figure 3B). To further analyze the grade of mosaicism, we performed deep sequencing analysis on DNA extracted from EDTA blood, urine and buccal swabs of individual 1. The variant was present in 29.65% of the 95,427 reads from the EDTA blood sample, in 35.64% of the 105,121 reads from urine, in 40.08% of the 97,698 reads from buccal swab (left side) and in 41.74% of the 108,035 reads from buccal swab (right side) (Figure 3C), suggesting a postzygotic occurrence of this variant. According to ACMG criteria (Richards et al., 2015) the variant was classified as likely pathogenic (PM2, PP2, PP3, and PM6) and submitted to ClinVar database (RCV001725829.4).

A trio ES strategy was performed on DNA extracted from blood of individual 2 (II-2) and both parents. Filtering for *de novo* variants identified the heterozygous splice variant in *STAG2* NM_001042749.2:c.1412_1416+9del p.?. Sanger sequencing was used to validate the NGS data for individual 2 and her parents. We could not detect the variant in the DNA extracted from peripheral blood lymphocytes of both unaffected parents (Figure 3B). The variant was not present in gnomAD and predicted to have a severe impact on protein function. According to *in silico* prediction the variant leads to a splice junction loss. We classified this variant as pathogenic according to ACMG criteria [(Richards et al., 2015); PVS1, PM2, and PP5] and submitted it to the ClinVar database (RCV001787300).

### 3.3 *STAG2* RNA sequencing and expression analyses

**FIGURE 4:**
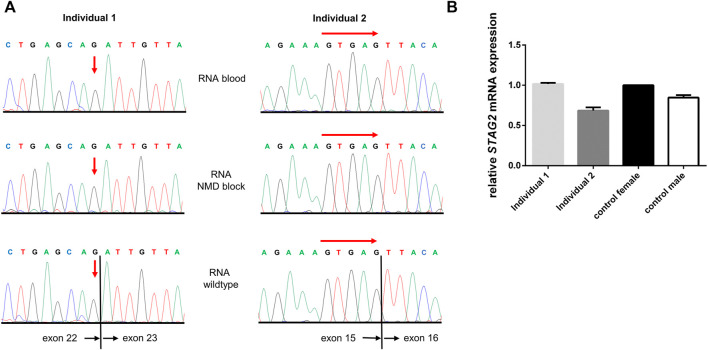
*STAG2* RNA sequencing and expression analyses. **(A)** Sequencing of *STAG2* transcripts for both individuals showed only wildtype sequences for the according regions. To block nonsense-mediated decay (NMD) lymphocytes from both individuals were treated with puromycin for 4 h and subsequently transcript analysis was performed. Again, no mutated or aberrantly spliced transcript was detected (middle panel). Red arrows indicate the locations of the expected variant (individual 1) or start of the expected deletion (individual 2). **(B)** Quantitative RT-PCR was performed for both individuals on RNA from blood, and relative *STAG2* expression of a healthy female control was set to 100% (black bar). Relative *STAG2* expression of individual 2 showed a reduction of *STAG2* expression to app. 60% (dark grey bar). In individual 1 the expression of *STAG2* was not reduced (light grey bar).

As both variants reported here were predicted to induce aberrant splicing by *in silico* prediction, we isolated RNA from peripheral blood lymphocytes of both affected individuals and analyzed *STAG2* pre-mRNA splicing by RT-PCR and subsequent Sanger sequencing of PCR fragments. Both sequence analyses only showed the correctly spliced *STAG2* wildtype (wt) transcript, and we were not able to detect any aberrantly spliced transcript in comparison to a wt control (Figure 4A). To further analyze whether nonsense-mediated mRNA decay (NMD) is the underlying cause of the absence of the aberrantly spliced allele, we treated blood lymphocytes with puromycin to suppress NMD. Subsequent RNA isolation, RT-PCR and Sanger sequencing, again, only revealed the correctly spliced *STAG2* transcripts. Furthermore, we quantified the expression of *STAG2* in both affected individuals by qPCR analysis on the RNA and showed that the expression of *STAG2* is reduced to approx. 60% in individual 2 (Figure 4B). In individual 1 the expression of *STAG2* was not reduced (Figure 4B). To exclude that the reduction in relative *STAG2* expression in individual 2 was based on primer-specific effects, we confirmed these results using a second primer pair for RT-PCR and were able to detect a similar reduction in relative *STAG2* mRNA expression (Supplementary Figure S1).

### 3.4 Transcriptome analyses during BENO generation by RNA sequencing

**FIGURE 5:**
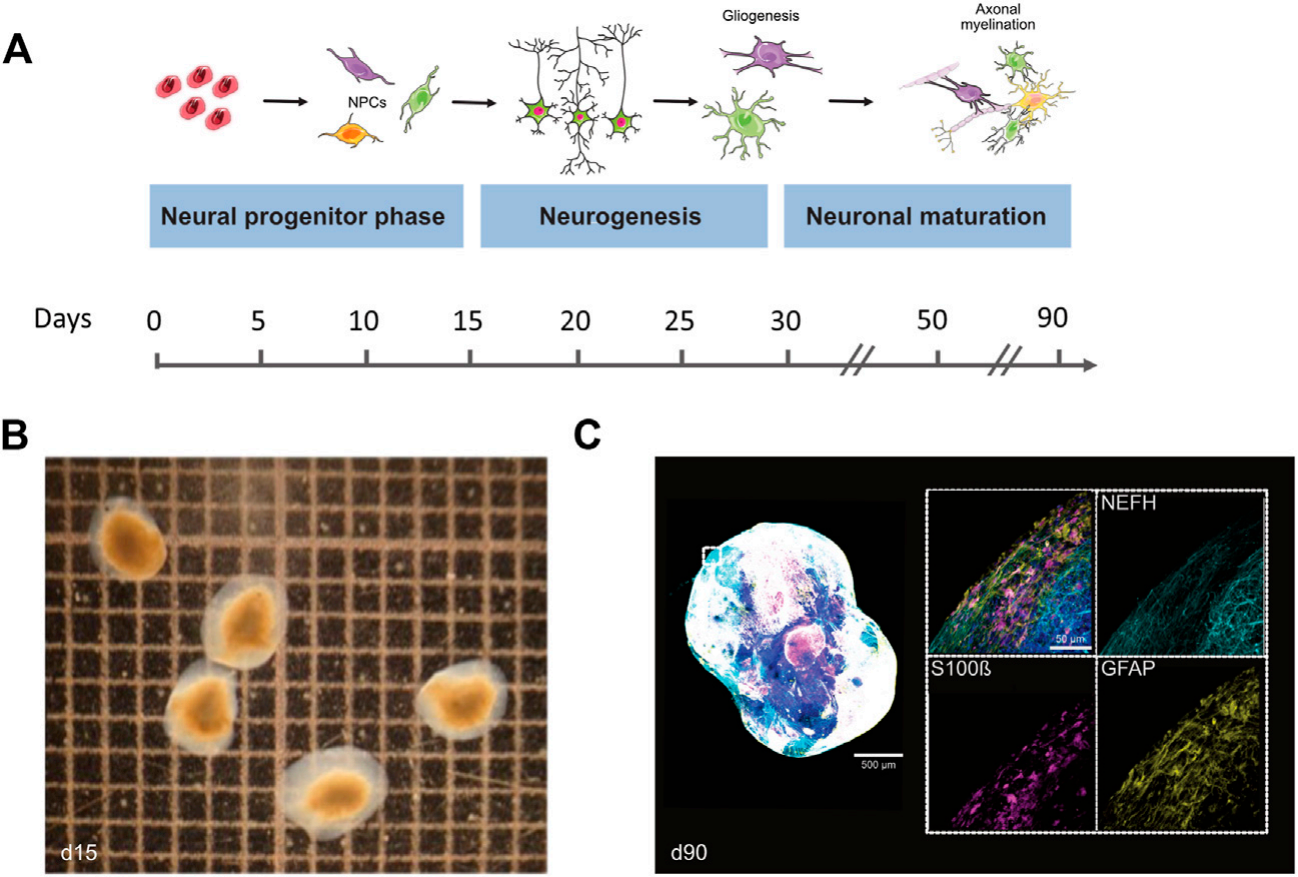
Differentiation and cellular composition of Bioengineered Neuronal Organoids (BENOs). **(A)** Scheme illustrates the different stages of BENO generation. Between days 0–10, neuroectodermal commitment is induced by dual smad inhibition (SB and LDN) and the presence of all-trans retinoic acid (RA). Neuronal progenitor cell growth is enhanced between days 10–15 by FGF-2 addition. Gliogenesis in BENOs is increased by addition of TGFß-1 at days 10–30. From day 15–30, DAPT is added to BENO cultures to promote neuronal differentiation. BENO development can be divided into four distinct stages: (1) Neuronal commitment to neuronal progenitor cells (NPCs) at days 3–15, (2) Neurogenesis between days 15–28, (3) Neuronal maturation from day 28 onwards and (4) Gliogenesis (day 50 onwards) such as axonal myelination (day 90 onwards). **(B)** Brightfield images of BENOs at day 15. **(C)** Representative whole mount immunofluorescence (WmIF) of BENOs from control hiPSC (TC1133, GMP) with astroglial markers GFAP and S100ß, and neuronal marker NEFH at day 90. Grid 1 × 1 mm. Graphics created with Servier Medical Art.

**FIGURE 6:**
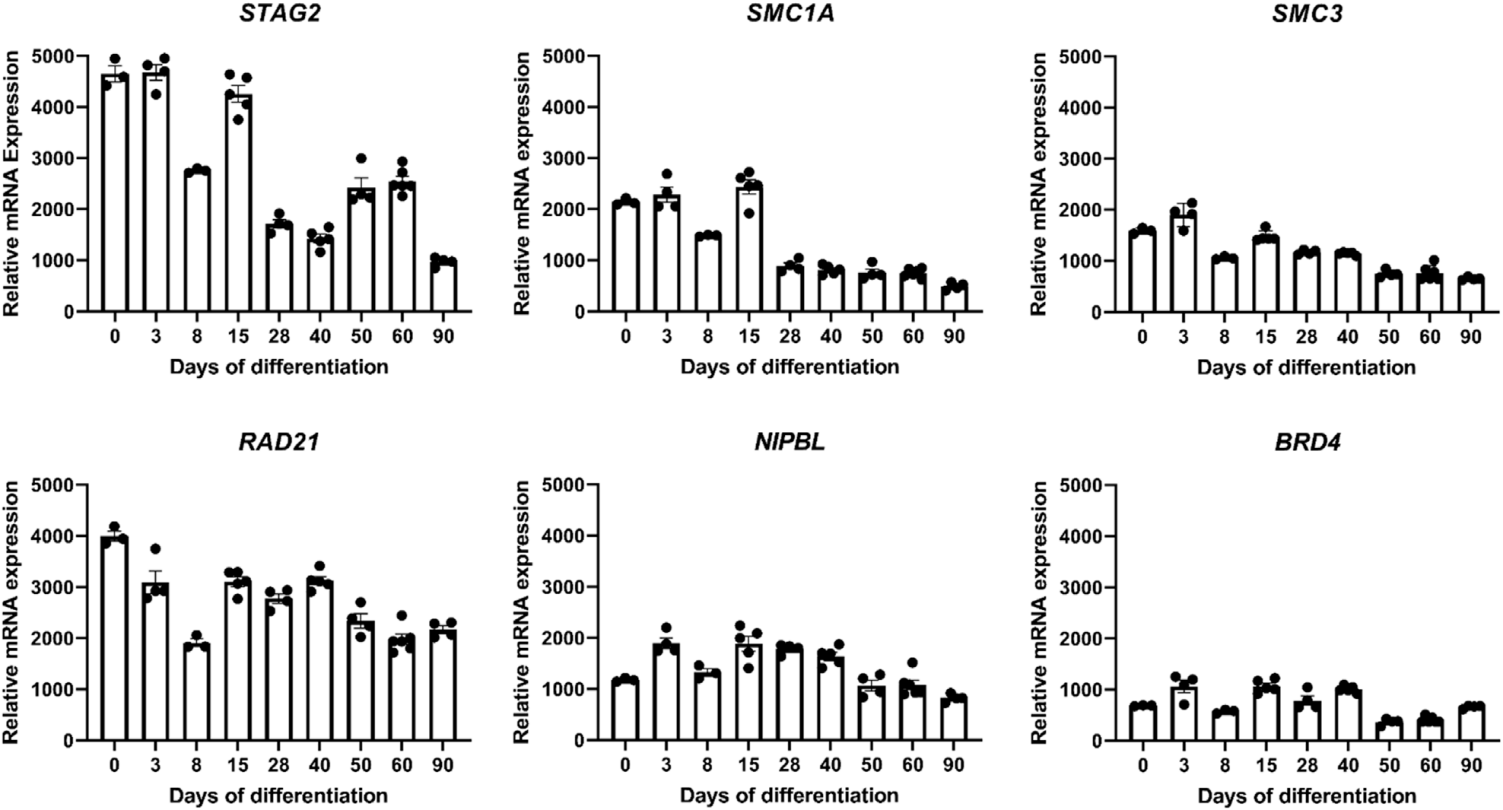
Transcriptome analyses during Bioengineered Neuronal Organoid (BENO) generation (from day 0 to day 90) by RNA sequencing. Transcriptome analysis of core subunits of the cohesin complex *STAG2, SMC1A, SMC3*, and *RAD21*, and interaction partner *NIPBL* that cooperates with *BRD4* during BENOs development (D0, undifferentiated iPSC; D3-15, neuroectoderm commitment; D28-40, neurogenesis; D50-90, gliogenesis and neuronal maturation).

In order to gain insights into the expression patterns of *STAG2* and associated genes during human brain development, we analyzed the expression level of *STAG2* and other components of the cohesin complex during Bioengineered Neuronal Organoids (BENOs) development (Figure 5) from human-induced pluripotent stem cells (iPSCs) by bulk RNA sequencing on culture days 0, 3, 8, 15, 28, 40, 50, 60, and 90 (Figure 6). BENOs are categorized as forebrain organoids consisting of excitatory and inhibitory neurons (Zafeiriou et al., 2020) as well as myelinating and non-myelinating glia (Figure 5C). Temporal transcript expression analysis showed that BENO undergo three sequential developmental processes (Figure 5A); neuronal commitment (d-0–15), neurogenesis (d-28-d-40), gliogenesis and neuronal maturation (d-50 onwards) (Zafeiriou et al., 2020). RNAseq data sets can be found at Gene Expression Omnibus (GEO) under the accession number GSE139101. T (d-0-d-60). D-90 data was submitted to the GEO and will be made available. *SMC1A, SMC3,* and *RAD21* were chosen as their gene products, like *STAG2*, are core subunits of the cohesin complex. NIPBL is an important cohesin interaction partner that cooperates with BRD4. We observed a prominent expression of all analyzed transcripts during BENO development (Figure 6). Notably, *STAG2* and *RAD21* presented a similar expression pattern and showed the higher expression especially during neuroectoderm commitment (between culture days 0 and 15), suggesting a critical role in forebrain development.

## 4 Discussion

In this study, we describe a mosaic *STAG2* variant in an individual with developmental delay, microcephaly, epilepsy, ataxia, and hemihypotrophy of the right side. Furthermore, we report an additional female with a *de novo* splice variant in *STAG2*. Of note, both individuals carry novel splice variants (Figure 3D). STAG2 is a component of the ubiquitously expressed, multi-subunit cohesin complex, which has been linked to various cellular processes (Peters and Nishiyama, 2012; Haarhuis et al., 2014; Hill et al., 2016). Numerous germline variants affecting subunits or regulators of the cohesin complex have previously been identified to cause a wide spectrum of multisystem developmental disorders belonging to the group of cohesinopathies (Parenti and Kaiser, 2021). CdLS, an autosomal dominant (*NIPBL, SMC3,* and *RAD21*) or X-linked (*SMC1* and *HDAC8*) malformation syndrome, represents the best characterized cohesinopathy (Kline et al., 2018). CdLS patients typically show pre- and post-natal growth retardation, intellectual disability, developmental delay, limb anomalies, and a characteristic craniofacial appearance including thick eyebrows, synophrys, a short nose with an upturned nasal tip, a long and smooth philtrum, and a thin upper lip (Kline et al., 2007; Kline et al., 2018). The majority of CdLS cases are caused by variants in the cohesin regulator *NIPBL* (Parenti and Kaiser, 2021; Chandrasekaran et al., 2022) with 15% of them predicted to affect splicing (Mannini et al., 2013). Noteworthy, mosaic variants are frequently (15%–20%) seen in individuals with a clinical diagnosis of CdLS (Krawczynska et al., 2018). In general, ultra-rare mosaic conditions can be challenging to deal with as, e.g., mosaic variants might be missed in the analysis of genomic DNA from standard peripheral blood samples. Therefore, a specific clinical suspicion of an underlying mosaic disorder might be required to select the appropriate tissue and to detect the causative variant. In some cases, the correct molecular diagnosis does not only allow accurate genetic counselling, but can also be highly relevant with regard to novel treatment options (Schmidt et al., 2022). Individual 1 reported here is the only patient described so far who carries a mosaic *STAG2* variant. In line with the genetic findings, individual 1 showed several manifestations typical of mosaic disorders, e.g., asymmetric disproportionate growth, dysplasia of the ear, hearing impairment, and supernumerary nipple affecting only the right side (Figure 1). Interestingly, both individuals reported here show supernumerary nipples, a feature which has not been reported to be associated to *STAG2* before. Consequently, we hypothesize that this is a further characteristic feature. Our findings indicate that mosaic *STAG2* variants should be considered as a cause of developmental delay, microcephaly, supernumerary nipples, and growth retardation, especially if features appear asymmetric or affect in particular one side of the body.

We performed deep sequencing analysis on DNA extracted from EDTA blood, urine, and buccal swabs of individual 1 to further analyze the grade of mosaicism. The *STAG2* variant c.2184=/G>T p.(Gln728=/His) was present in 29.65% of the 95,427 reads from the EDTA blood sample, in 35.64% of the 105,121 reads from urine, in 40.08% of the 97,698 reads from the buccal swab (left side) and in 41.74% of the 108,035 reads from the buccal swab (right side) (Figure 3C), suggesting a postzygotic occurrence. For individuals with the tentative diagnosis of CdLS and negative genetic test results, it was proposed that *NIPBL* and other CdLS genes should be tested for mosaicism using DNA extracted from buccal cell swabs, skin fibroblasts, or bladder epithelial cells from urine (Kline et al., 2018). Based on our results, we suggest the same testing strategy for *STAG2*.

To verify the consequences of both *in silico* predicted splice variants in the reported individuals, we performed mRNA sequencing of *STAG2* transcripts. Surprisingly, we were not able to detect the mutated or aberrantly spliced *STAG2* transcripts in neither individual, even after blockage of NMD using puromycin (Figure 4A). Aoi et al. (2020) reported a similar result as in our cases. Using RT-PCR sequencing in a female carrying a *de novo* nonsense variant in *STAG2*, they only detected wt *STAG2* transcripts, even after blockage of NMD by cycloheximide treatment (Aoi et al., 2020). In our study, however, mRNA expression analysis in individual 2 showed a reduced *STAG2* expression in blood lymphocytes, whereas the expression of *STAG2* was not reduced in individual 1 (Figure 4B). This might be explained by the grade of mosaicism in blood cells combined with skewed X-inactivation and a tissue-specific expression pattern of *STAG2*. In addition, the *STAG2*-associated phenotype together with the expression data generated by transcriptome analyses during BENO development might indicate that *STAG2* is especially expressed during a specific period of the development during embryogenesis. In line with our results from *STAG2* transcript sequencing and expression analysis this points to pre-transcriptional repression of mutated *STAG2* transcript rather than post-transcriptionally activated NMD. Of note, all experiments were performed in (cultured) lymphocytes, which might not represent identical events of *STAG2* transcript as occurring in the affected tissues of individuals.

Our clinical findings in line with previously published reports indicate that the *STAG2*-associated phenotype is dominated by global developmental delay, severe microcephaly, and brain abnormalities. Importantly, cMRI images of both individuals with *STAG2*-associated cohesinopathy presented here showed profound brain anomalies, e.g., delayed and incomplete myelination, hypotrophy, thin and dysplastic corpus callosum, or semilobar holoprosencephaly (Figure 2). Therefore, our observations further support that loss-of-function variants in *STAG2* seem to cause especially midline brain malformations, including holoprosencephaly, agenesis of the corpus callosum, and dysgenesis of the corpus callosum (Kruszka et al., 2019). A previous study has shown that *STAG2* is expressed in the neural fold at the critical time of forebrain division in mice (Kruszka et al., 2019). Morphogenesis of the brain is regulated by several complex biological pathways and the precise mechanism of abnormal forebrain development caused by loss-of-function variants in *STAG2* is still unknown. Brain organoids have been introduced as promising tools to gain further insights into the impaired development of the human nervous system (Lancaster et al., 2013; Zafeiriou et al., 2020; Islam et al., 2021; Wrobel et al., 2021; Tang et al., 2022). In our recently established novel Bioengineered Neuronal Organoids (BENOs) key aspects of human brain development (Figure 5), such as neuronal network formation with demonstrated network activity (plasticity, long- and short term potentiation) could be demonstrated for the first time (Zafeiriou et al., 2020). To test whether BENOs are also a suitable model for *STAG2*-associated cohesinopathies, we performed RNA sequencing to investigate the expression of *STAG2* and other core components and interaction partners of the cohesin complex during BENO development (Figure 6). We observed a strong expression of *SMC1A, SMC3*, *RAD21, NIPBL* and *BRD4* during BENO development. Notably, *STAG2* and *RAD21* showed the highest expression during neuronal commitment in BENO (between culture days 0 and 15), in line with the expression found in the neural fold during forebrain development in mice (Kruszka et al., 2019). Given our RNA expression data, we hypothesize that *STAG2* and *RAD21* play a critical role in early brain development. Loss-of-function variants in RAD21 can cause CdLS4 (Deardorff et al., 2012b). Midline brain anomalies were also detected in association with CdLS4 (Goel and Parasivam, 2020; Kruszka, 2020). Further experimental investigations are necessary for a better molecular understanding of how the cohesin complex regulates brain development. Towards this end, we anticipate that BENOs with all critical components expressed represent an attractive tool to gain further insights into the critical role of STAG2 during the complex development of the nervous system. Interestingly, a recently published study analyzed conditional knockout mice with Stag2 deleted in the nervous system (Cheng et al., 2022). Cheng et al. (2022) showed that *Stag2* ablation downregulates the expression of myelin genes in mouse brains and compromises myelination during early postnatal development. In addition, their findings provide evidence that *STAG2* generates promoter-anchored loops at myelination-promoting genes to facilitate their transcription.

Together, our findings indicate that mosaic *STAG2* variants should be considered as a cause of developmental delay, microcephaly, supernumerary nipples, and growth retardation and we expand the complex genotypic and phenotypic spectrum of cohesinopathies. For a better understanding of the molecular consequences caused by loss-of-function variants in *STAG2*, investigation of modified BENOs appears to be a highly promising approach.

## Data Availability

The datasets presented in this study can be found in online repositories. The names of the repository/repositories and accession number(s) can be found below: https://www.ncbi.nlm.nih.gov/geo/, GSE139101.
