# Supplementary material for: Somatic mosaicism in STAG2-associated cohesinopathies: Expansion of the genotypic and phenotypic spectrum
Source: Front Cell Dev Biol. 2022 Nov 16;10:1025332. doi: 10.3389/fcell.2022.1025332 (PMC9710855; doi:10.3389/fcell.2022.1025332)
Supplement: Supplementary file 2 [file Table1.DOCX]

Table S1. Primers used for Sanger sequencing and transcriptome analysis

| Primer name | Sequence |
| --- | --- |
| STAG2_cDNA_qPCR_2F | CAGCTTTGGTGAATGTGGCA |
| STAG2_cDNA_qPCR_2R | AGCCTCTCATTGGCTCGTTT |
| STAG2_cDNA_qPCR_4F | GGCATGCAGCTTTCACTCAC |
| STAG2_cDNA_qPCR_4R | CTGTGTTGGCGTACAGGTCT |
| ACTB_cDNA_qPCR_F | CTTCCAGCCTTCCTTCCTGG |
| ACTB_cDNA_qPCR_R | CTGTGTTGGCGTACAGGTCT |
| Li_STAG2-Ex22F | CAGCTCTCCCACAGGCGAATGCAGTGCGTGAATAACAATctg |
| Li_STAG2-Ex22R | GGTCAGCTCTCCCAGCGATatttgttgttgtcgttgtgggg |
| Li_STAG2-cDNA-Ex20F1 | GGTCAGCTCTCCCAGCGATTGCCTTATTGCGACAGATCCGG |
| Li_STAG2-cDNA-Ex21F2 | GGTCAGCTCTCCCAGCGATGTGAAGAACCTGATGAAGATGATGC |
| Li_STAG2-cDNA-Ex23R2 | CAGCTCTCCCACAGGCGAACAAGTTGCCAAAGGATTACATAGTGAG |
| Li_STAG2-cDNA-Ex24R1 | CAGCTCTCCCACAGGCGAAACAGTAGTATTCACGTTGGTCAGG |
| Li_STAG2-Ex15F | GGTCAGCTCTCCCAGCGATacctacccagttctcctttactcc |
| Li_STAG2-Ex15R | CAGCTCTCCCACAGGCGAAtgtgcctcattttaaccccttttcc |
| Li_STAG2-cDNA-Ex12F1 | GGTCAGCTCTCCCAGCGATTGCATGATAAGCAAGGTGAAGT |
| Li_STAG2-cDNA-Ex18R1 | CAGCTCTCCCACAGGCGAACTCCTTTGCTGTAAGCACCC |
| Li_STAG2-cDNA-Ex12F2 | GGTCAGCTCTCCCAGCGATTGTCTTACTGCTCTACAAGGGC |
| Li_STAG2-cDNA-Ex18R2 | CAGCTCTCCCACAGGCGAACCACGGCAAAAAGCTCAGTG |
| Li_STAG2-cDNA-Ex14F1 | GGTCAGCTCTCCCAGCGATTGGTTTATTCAGCTCACCGGCC |
| Li_STAG2-cDNA-Ex16R1 | CAGCTCTCCCACAGGCGAATCCCAGTCTTTCAGCAGCTCAG |
