# Supplementary material for: Somatic mosaicism in STAG2-associated cohesinopathies: Expansion of the genotypic and phenotypic spectrum
Source: Front Cell Dev Biol. 2022 Nov 16;10:1025332. doi: 10.3389/fcell.2022.1025332 (PMC9710855; doi:10.3389/fcell.2022.1025332)
Supplement: Supplementary file 3 [file DataSheet1.docx]

**Supplementary Figure S1.** Relative *STAG2* expression of individual 2 showed a reduction of *STAG2* expression to app. 50% with both different primer pairs used. Quantitative RT-PCR was performed for individual 2 on RNA from blood, and relative *STAG2* expression of a healthy female control was set to 100%.
